# Cerebrovascular Complications and Vessel Wall Imaging in COVID-19 Encephalopathy—A Pilot Study

**DOI:** 10.1007/s00062-021-01008-2

**Published:** 2021-03-26

**Authors:** Marjolaine Uginet, Gautier Breville, Jérémy Hofmeister, Paolo Machi, Patrice H. Lalive, Andrea Rosi, Aikaterini Fitsiori, Maria Isabel Vargas, Frederic Assal, Gilles Allali, Karl-Olof Lovblad

**Affiliations:** 1 Division of Neurology, Clinical Neurosciences Department, Geneva University Hospitals, Geneva, Switzerland; 2 Division of Neuroradiology, Diagnostic Department, Geneva University Hospitals, 1211 Geneva, Switzerland; 3 Department of Neurology, Division of Cognitive and Motor Aging, Albert Einstein College of Medicine, Yeshiva University, Bronx, NY USA

**Keywords:** Inflammation, MRI, Contrast agent, Stroke, Diffusion, SWI

## Abstract

**Background and Purpose:**

Coronavirus disease 2019 (COVID-19) caused by the severe acute respiratory syndrome coronavirus 2 (SARS-CoV-2) is associated with several complications of the central nervous system (CNS), including acute encephalopathy.

**Methods:**

In this pilot study, we report a series of 39 patients (66.5 ± 9.2 years; 10.3% female) with acute encephalopathy, who underwent a standard brain magnetic resonance imaging (MRI) at 1.5 T during the acute symptomatic phase. In addition to diffusion-weighted imaging, MR angiography and susceptibility-weighted images, high-resolution vascular black blood sequences (in 34 cases) were used to investigate the vasculature of the brain.

**Results:**

In 29 out of 34 patients with COVID-19 encephalopathy (85%) with high-resolution vessel wall imaging, we found a circular enhancement and thickening of the basilar and vertebral arteries, without any correlation with ischemia or microbleeds (reported in 21% and 59%, respectively).

**Conclusion:**

We report a high prevalence of vascular changes suggestive of endotheliitis as reported in other organs. This could suggest an inflammatory mechanism underlying this encephalopathy*.*

## Introduction

Coronavirus disease 2019 (COVID-19), which has spread all over the globe causing mainly respiratory symptoms, has been associated with extrapulmonary manifestations [1], including idiopathic encephalopathy—the COVID-19 encephalopathy [2]. In the vast majority of reports, no direct proof of the virus has been demonstrated in the cerebrospinal fluid (CSF) [3, 4] leading to the hypothesis of an immune or an inflammatory mechanism. Neuroradiological reports describe various neurological complications, such as cerebrovascular diseases including stroke and microbleeds associated with COVID-19 [5, 6]. Severe COVID-19 patients with stroke are characterized by a hyperinflammatory status leading to a prothrombotic state [7]. Cases of vasculitis with or without stroke have also been reported [5, 8]. Similar to other organs, neuropathological findings demonstrated the presence of vascular inflammation of the endothelium [2, 9]. The hypothesis of an endotheliitis (due to the so-called cytokine storm or via the angiotensin-converting enzyme 2 present on the vascular wall) for this encephalopathy has been raised due to the observed vascular patterns in some cases. This suggests that investigating the brain vasculature with modern Magnetic Resonance (MR) techniques is the next logical step. Thus, to test the endothelial hypothesis supporting this acute encephalopathy, we systematically used black blood vessel wall imaging among patients with COVID-19 encephalopathy.

## Material and Methods

### Patients and Study Design

This was a retrospective observational study, performed at our institution between 16 March and 22 May 2020. The study has been accepted by the local ethics committee who gave a consent waiver (protocol #2020-01206). We included 39 consecutive adult patients with COVID-19 encephalopathy (35 men, 4 women, age: 66.5 ± 9.2 years) admitted to our institution and tested positive for severe acute respiratory syndrome coronavirus 2 (SARS-CoV-2). All these patients underwent a brain MRI due to an acute encephalopathy: 20 were comatose and 19 presented with delirium. Encephalopathy was defined by a rapidly developing (less than 4 weeks) pathobiological process in the brain leading to delirium or coma without any identified etiology, after appropriate screening and exclusion of classical medical etiologies, such as electrolyte disturbances, infections, drug or alcohol toxicity and/or withdrawal, metabolic disorders (e.g. renal failure), or low perfusion state. Additional exclusion criteria included all reported causes of neurological complications of COVID-19, such as Guillain-Barré syndrome, posterior reversible encephalopathy syndrome, venous sinus thrombosis or necrotizing encephalopathy; also there was no autoimmune disease associated.

### Imaging Technique and Evaluation

The MR images were acquired on a 1.5 T clinical scanner (Philips Ingenia (Philips Medical Systems, Eindhoven, The Netherlands)) equipped with a head and neck coil. In addition to a standard neuro head protocol comprising axial T1 and T2-weighted, axial diffusion-weighted imaging (DWI), susceptibility-weighted images (SWI) for the detection of blood, as well as 3D time-of-flight (TOF) MR angiography (MRA) of the intracranial vessels, a dynamic 3D contrast-enhanced MRA of the neck vessels was performed from the aortic arch to the circle of Willis. Precontrast and postcontrast fat-saturated T1-weighted black blood VISTA images in all patients (TE: 17 ms, TR: 400 ms, image thickness 1.5 mm) were acquired in the axial and coronal planes. Measurement of the longitudinal length of the arterial wall enhancement was manually performed on the injected T1-weighted MR images.

All MRI were blindly reviewed by three board-certified neuroradiologists and contrast vessel enhancement was validated when common agreements were reached. Additionally, they looked for the presence of hypointensities on SWI as signs of microbleeds, and for ischemia on the diffusion-weighed MR images at the high b value, seen as hyperintensities. The MR angiographic images were evaluated for the presence of arteriosclerotic changes.

### Statistics

Baseline characteristics were summarized using means and standard deviations. Between-group comparisons (ischemia versus no ischemia; and microbleeds versus no microbleeds) were performed using unpaired t‑test, Mann-Whitney U‑test or Fisherʼs exact test, as appropriate. All analyses were conducted using SPSS version 25 (SPSS Inc., Chicago, IL, USA).

## Results

Of the 39 patients undergoing MRI, 34 patients underwent the full protocol including contrast-enhanced (CE) magnetic resonance imaging of the brain with CE-MRA of the neck vessels as well as high-resolution vessel wall imaging using T1-weighted fat saturated VISTA images.

### Vessel Wall Enhancement

**Fig. 1:**
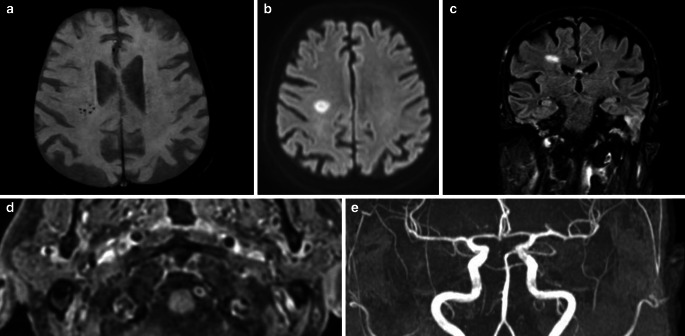
A 73-year-old patient, without comorbidity, hospitalized with severe SARS-CoV‑2 infection having required intubation (26 days) and presenting delayed awakening, after withdrawing all sedative medication and no clear etiology despite extensive work-up. Magnetic resonance imaging: axial SWI shows microbleeds in the right deep white matter (**a**). axial DWI shows an acute lesion in the same area (**b**) along with edema on coronal Fluid-attenuated inversion recovery (FLAIR) (**c**). T1 postcontrast axial black blood VISTA images in this patient (**d**) (TE: 17 ms, TR: 400 ms, image thickness 1.5 mm); image shows circumferential enhancement in the wall of the left vertebral artery. No stenosis in the affected vessel on the coronal projection TOF MRA (**e**)

**Fig. 2:**
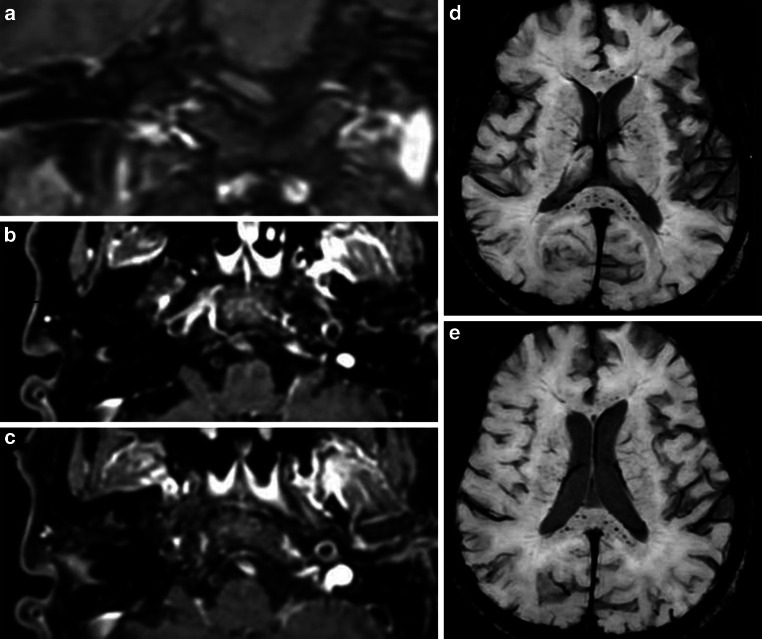
A 62-year-old patient hospitalized for acute respiratory distress syndrome due to SARS-CoV‑2 infection with intubation over 13 days. This patient was overweight and had mild cognitive impairment. He had developed a persistent delirium over more than 10 days after withdrawal of all drugs and without focal neurological deficit. MRI shows strong enhancement of the left vertebral artery (**a–c**), SWI images show microbleeds, preferentially in the corpus callosum (**d,** **e**)

**Fig. 3:**
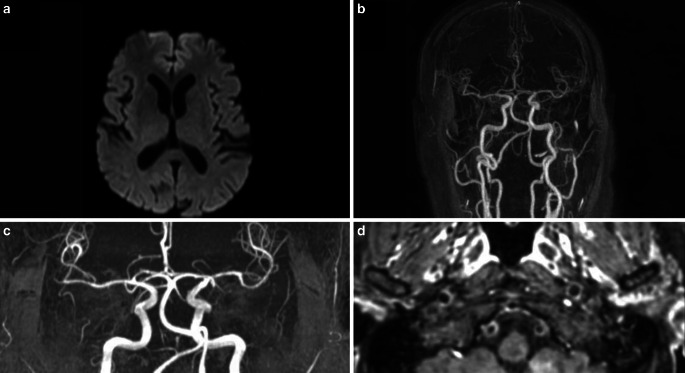
A 61-year-old patient hospitalized for dyspnea due to SARS-CoV‑2 infection with intubation over 10 days. His medical history included ischemic heart disease and sleep apnea syndrome, but no psychiatric or cognitive impairment. He had a persistent and unexplained delirium persisting for 12 days after stopping all sedative drugs, without neurological deficit. Magnetic resonance imaging: no changes on DWI (**a**). Absence of stenosis on CE-MRA (**b**) and TOF MRA (**c**) Bilateral enhancement of the vertebral arteries on black blood VISTA images (TE: 17 ms, TR: 400 ms, image thickness 1.5 mm) (**d**)

Out of the 34 patients 29 (85%) had a circumferential enhancement of the intracranial vertebral and basilar arteries on the VISTA images. The five remaining had a full head protocol with no contrast. The length of the enhancement varied between 4.00 mm and 42.00 mm (18.00 ± 9.76 mm). The enhancement was unilateral in 12 cases (35%)> (Fig. 1 and 2)  and bilateral at the level of the vertebral arteries in 17 patients (50%) (Fig. 3) and, in the unilateral cases, more often on the left ([10]; Fig. 1), with only 2 cases having unilateral right-sided enhancement. No evident stenosis was reported on the associated TOF MRA or on the CEMRA. The length of the enhancement was similar between patients with and without ischemia. The severity of the encephalopathy (measured by the CAM [Confusion assessment method] scale) was not associated with the length of the enhancement (r = −0.108; *p*-value = 0.551).

### Ischemic Lesions

Table 1 compares clinical and radiological characteristics of COVID-19 encephalopathy patients with and without ischemia. On DWI, 8/39 (21%) patients had acute ischemic stroke (Fig. 1). In all cases, the acute lesions were not associated with neurological symptoms; however, the neurological examination can be particularly difficult in comatose patients. Our comatose patients had had a wake-up test (interruption of sedation) whereupon no neurological deficits were noted. In 4 patients with ischemia (50%), the lesions were smaller than 5 mm. Moreover, the acute lesions were not in the involved vascular territory of the vessel enhancement. Age, cardiovascular risk factors and chronic cardiac diseases were similar between patients with and without ischemic lesions.

**Table 1:** Comparison of COVID-19 encephalopathy patients with and without stroke

	Total (*n* = 39)	Ischemia (*n* = 8)	No ischemia (*n* = 31)	*P*-value
Age (years)	66.5 ± 9.2	67.1 ± 8.9	66.3 ± 9.4	0.843
Gender *n* (% female)	4 (10.3)	2 (25)	2 (6.5)	0.180
Education level, mean (on a scale 0–3)	2.33 ± 0.76	2.43 ± 0.53	2.30 ± 0.82	1.000
Duration of COVID-19 symptoms before SACRE onset (days)	19.3 ± 9.6	15.4 ± 16.7	20.0 ± 8.2	0.573
Intubation (days) (*n* = 36)	14.3 ± 5.7	11.7 ± 9.2	15.0 ± 4.5	0.850
Duration of SACRE before MRI (days)	10.8 ± 10.9	12.9 ± 8.0	10.3 ± 9.5	0.509
**Comorbidities *n* (%)**				
Smoking	3 (7.9)	0 (0)	3 (10.0)	0.481
Cardiovascular risk factor^a^ (0–4)	29 (74.4)	7 (87.5)	22 (71.0)	0.323
Body mass index (kg/m^2^)	28.7 ± 5.6	30.5 ± 5.3	28.3 ± 5.7	0.339
Chronic cardiac disease^b^	13 (33.3)	4 (50.0)	9 (29.0)	0.238
Pulmonary diseases^c^	7 (17.9)	3 (37.5)	4 (12.9)	0.137
Dementia^d^	4 (10.5)	1 (12.5)	3 (10.0)	0.629
**Neurological signs at neurological evaluation *n* (%)**				
CAM (total score)	2.74 ± 1.07	3.00 ± 1.41	2.67 ± 1.00	0.068
RASS (total score)	−1.00 ± 1.82	−1.25 ± 1.83	−0.94 ± 1.84	0.669
Focal neurological signs	10 (26.3)	2 (25.0)	8 (26.7)	0.652
Corticospinal tract signs	16 (43.2)	3 (37.5)	13 (44.8)	0.517
Sensory deficit	3 (10.3)	1 (14.3)	2 (9.1)	0.579
Cranial nerve deficit	5 (12.8)	1 (12.5)	4 (12.9)	0.732
**MRI**				
*White matter abnormalities (0–3)*	1.18 ± 1.05	1.75 ± 0.89	1.03 ± 1.04	0.483
*Microbleeds n, (%)*	23 (59.0)	6 (75.0)	17 (54.8)	0.269
*Vessel enhancements n, (%)*	29 (87.9)	5 (83.3)	24 (88.9)	0.571
Concentric enhancements	24 (82.8)	4 (80.0)	20 (83.3)	0.642
Length vessel enhancements (mm)	18.00 ± 9.76	20.4 ± 12.6	17.5 ± 9.3	0.555
Unilateral/bilateral enhancements	12/17	3/2	9/15	0.329

*COVID-19* coronavirus disease 2019, *CAM* confusion assessment methods, *RASS* Richmond agitation sedation scale, *SACRE* SARS COVID related encephalopathy

^a^Cardiovascular risk factors: hypertension; diabetes, dyslipidemia, obstructive sleep apnea

^b^Chronic cardiac disease: coronary artery disease or congestive heart failure

^c^Pulmonary diseases: chronic obstructive pulmonary disease or interstitial lung disease

^d^Dementia: chronic neurodegenerative disease or vascular dementia

### Microbleeds

Clinical and radiological comparisons of COVID-19 encephalopathy patients with and without microbleeds (and their distribution) are presented in Table 2. On SWI, 23/39 (59%) patients had microbleeds (Fig. 2). The repartition of the microbleeds was not systematic: deep in 6/23 (26%), superficial in 8/23 (34.8%) and mixed in 9/23 (31.1%). Similar to ischemia, there was no association with vessel enhancement or acute ischemia. The majority of patients presented various microbleeds: more than 3 microbleeds in 16/23 (70%) and more than 10 microbleeds in 5/23 (21.7%). The clinical presentation, especially focal neurological signs, was similar between patients with and without microbleeds. The clinical presentation, including vascular risk factors and premorbid dementia, were similar between patients with and without microbleeds.

**Table 2:** Comparison of COVID-19 encephalopathy patients with and without microbleeds

	Total (*n* = 39)	Microbleeds (*n* = 23)	No microbleeds (*n* = 16)	*P*-value
Age (years)	66.5 ± 9.2	67.7 ± 8.7	64.8 ± 1	0.344
Gender (% female)	4 (10.3)	1 (4.3)	3 (18.8)	0.179
Intubation duration (days)	14.3 ± 5.7	11.7 ± 6.7	15.5 ± 6.7	0.082
**Comorbidities *n* (%)**				
Smoking	3 (7.9)	3 (13.0)	0 (0.0)	0.210
Cardiovascular risk factor (0–4)	29 (74.4)	17 (73.9)	12 (75.0)	0.620
Body mass index (kg/m^2^)	28.7 ± 5.6	27.8 ± 4.7	30.1 ± 6.6	0.226
Chronic cardiac disease	13 (33.3)	8 (34.8)	5 (31.3)	0.548
Pulmonary diseases	7 (17.9)	6 (26.1)	1 (6.3)	0.121
Dementia	4 (10.5)	3 (13.0)	1 (6.7)	0.480
**Neurological signs at neurological evaluation *n* (%)**				
CAM (total score)	2.74 ± 1.07	2.61 ± 1.23	2.93 ± 0.77	0.861
RASS (total score)	−1.00 ± 1.82	−0.91 ± 1.92	−1.12 ± 1.71	0.726
Focal neurological signs	10 (26.3)	8 (34.8)	2 (13.3)	0.137
Corticospinal tract signs	16 (43.2)	8 (36.4)	8 (53.3)	0.247
Cranial nerve deficit	5 (12.8)	3 (13.0)	2 (12.5)	0.674
Sensory deficit	3 (10.3)	3 (16.7)	0 (0.0)	0.223
**Distribution of cerebral microbleeds**				
Number, *n*	–	10.0 ± 20.4	–	–
Deep, *n* (%)	–	6 (26.1)	–	–
Superficial, *n* (%)	–	8 (34.8)	–	–
Mixed, *n* (%)	–	9 (31.1)	–	–
Corpus callosum, *n* (%)	–	7 (30.4)	–	–
**MRI**				
White matter abnormalities (0–3)	1.18 ± 1.05	1.17 ± 1.07	1.19 ± 1.05	0.908
Acute stroke, *n*	8 (20.5)	6 (26.1)	2 (12.5)	0.269
Vessel enhancements (%)	29 (87.9)	18 (85.7)	11 (91.7)	0.536
Length vessel enhancements (mm)	18.00 ± 9.76	19.11 ± 9.58	16.18 ± 10.24	0.443

*CAM* confusion assessment methods, *RASS* Richmond agitation sedation scale

### CSF Analysis

The CSF white blood cell count was normal in 15/16 patients (mean value: 1.94 ± 2.08 M/L). The CSF/serum quotient of albumin (QAlb measured in 13 patients) was increased in 69.2% (mean QAlb = 11.6 ± 6.9). The presence of oligoclonal bands type 4 was reported in 11/13 patients (84.6%) and the presence of oligoclonal bands type 5 in 1/13 patients (7.6%), indicating no intrathecal IgG synthesis in the CSF. No specific intrathecal IgG synthesis (oligoclonal bands type 2 or 3) was observed in the 13 patients with available CSF.

## Discussion

In this series of patients with COVID-19, we focused on patients with acute encephalopathy. A large majority of these patients with COVID-19 encephalopathy presented a gadolinium vessel enhancement mostly at the level of the basilar and intracranial vertebral arteries seen on the black blood fat saturated T1-weighted images. Additionally, in these COVID-19 patients with encephalopathy we reported an increased prevalence of microbleeds and ischemia but they were not related to any specific neurological symptoms. Even if the neurological examination is difficult, sedation was lifted in order to assess the patients’ status [10, 11]*.*

Patients with COVID-19 encephalopathy showed an increased prevalence of gadolinium vessel enhancement. Previous studies in COVID-19 patients have also reported a similar brain vascular enhancement but in a smaller proportion [6, 8]. The extremely high proportion of vessel enhancement reported in the current study may be explained by the selection of COVID-19 patients with encephalopathy. Interestingly, the vascular enhancement affects more frequently the left vertebral artery than the right; this left predominance may be due by the observation that the left vertebral artery is usually the dominant artery in 60% of healthy individuals [12]. Therefore, this vascular enhancement could support the hypothesis of an underlying inflammatory origin of COVID-19 encephalopathy [6]. This inflammatory hypothesis is also supported by a previous study showing that 11/19 (58%) patients with neurological manifestations due to COVID-19 demonstrated signs of blood-brain barrier (BBB) disruption [3]. Similarly, in our subsample of patients with CSF analysis, we observed an increased QAlb in 69.2%, suggestive of a BBB disruption. The BBB disruption could be induced by a direct effect of SARS-CoV‑2, or secondary to a disproportionate inflammatory reaction induced by SARS-CoV‑2 leading to an endotheliitis [9]. Furthermore, a recent neuropathological study showed endotheliitis in a series of COVID-19 patients [13].

More than 20% of ischemic lesions are reported in this series of COVID-19 patients with acute encephalopathy; a large majority of ischemic lesions of small size without clinical expression. Cardiovascular risk factors (74.4%), including obesity and chronic cardiac disease were reported in the majority of patients; however, patients with and without ischemic lesions were similar in terms of comorbidities, such as cardiovascular risk factors or cardiac diseases, suggesting a specific COVID-19-related mechanism that may be associated to a hyperinflammatory status; however due to the morphology of the ischemic lesions we cannot exclude an atherosclerotic mechanism associated with these lesions. Even though not present in our series, a few cases of neck artery dissection have been reported in COVID-19 patients and we cannot exclude that the suspected inflammation of the vessel wall could pave the route for a vascular vulnerability that would cause a dissection [14, 15]*.*

Cerebral microbleeds are reported in the majority of patients with COVID-19 encephalopathy, exceeding the prevalence of microbleeds reported in COVID-19 patients [2, 6, 16] including those hospitalized in the intensive care unit [17]. This increased prevalence of microbleeds reported in the current series can be explained by the strict inclusion of patients with COVID-19 encephalopathy. This COVID-19 encephalopathy may be secondary to an inflammatory mechanism of the vessel wall leading to a BBB disruption [2, 6]; a similar hypothesis has been suggested in patients with microbleeds due to amyloid angiopathy [18]. The pattern of the microbleeds distribution is unspecific in patients with COVID-19 encephalopathy and does not follow those reported in patients with amyloid angiopathy [19], in patients with hypertension [20] or in patients with critical illnesses [21]. Hypoxemia and high concentration of uremic toxins leading to BBB dysfunction in patients with COVID-19 have been suggested as the main mechanism leading to cerebral microbleeds in COVID-19 and may explain this atypical pattern of distribution and this increased prevalence of microbleeds [22].

Including a high number of patients with COVID-19 encephalopathy with high-resolution vascular sequences represent the main strength of this study; however, this study is not without limitations. All patients were hospitalized and the majority of them (82%) has been intubated, preventing the generalization of the study findings. The support of blood and CSF analyses, especially cytokines profile, would have strengthen the pathophysiological hypothesis. We should acknowledge that all patients did survive during hospitalization, suggesting that the patients with the most severe COVID-19 (those who eventually died) were not referred for a neurological consultation and subsequently not included in this series that also restricts the generalization of the study findings. There were therefore no neuropathological findings. Finally, although neuropathological findings did also support the hypothesis of an endotheliitis for this encephalopathy [2], the comparison with a control group of patients with COVID-19 without encephalopathy would have reinforced the hypothesis of an endotheliitis for explaining the COVID-19 encephalopathy.

In conclusion, cerebrovascular complications, such as ischemia and microbleeds, are frequent in patients with COVID-19 encephalopathy; a large majority of these patients show a gadolinium vessel enhancement suggestive of an endotheliitis. Future studies should include a detailed evaluation of inflammatory markers to support this inflammatory pathophysiological mechanism.
